# Music Use for Sedation in Critically ill Children (MUSiCC trial): a pilot randomized controlled trial

**DOI:** 10.1186/s40560-020-00523-7

**Published:** 2021-01-12

**Authors:** Gonzalo Garcia Guerra, Ari R. Joffe, Cathy Sheppard, Krista Hewson, Irina A. Dinu, Morteza Hajihosseini, Allan deCaen, Hsing Jou, Lisa Hartling, Sunita Vohra, the Canadian Critical Care Trials Group

**Affiliations:** 1 Department of Pediatrics, University of Alberta, Room 4-548 11405 87 Avenue, Edmonton, AB T6G 1C9 Canada; 2 Stollery Children’s Hospital, Edmonton, AB Canada; 3 Department of Educational Psychology, University of Alberta, Edmonton, AB Canada; 4 School of Public Health, University of Alberta, Edmonton, AB Canada; 5 Integrative Health Institute, University of Alberta, Edmonton, AB Canada

**Keywords:** Sedation, Analgesia, Music, Noise, Pediatric intensive care

## Abstract

**Objective:**

To demonstrate feasibility of a music medicine intervention trial in pediatric intensive care and to obtain information on sedation and analgesia dose variation to plan a larger trial.

**Material and methods:**

Pilot randomized controlled trial (RCT) was conducted at the Stollery Children’s Hospital general and cardiac intensive care units (PICU/PCICU). The study included children 1 month to 16 years of age on mechanical ventilation and receiving sedation drugs. Patients were randomized in a 1:1:1 ratio to music, noise cancellation or control. The music group received classical music for 30 min three times/day using headphones. The noise cancellation group received the same intervention but with no music. The control group received usual care.

**Results:**

A total of 60 patients were included. Average enrollment rate was 4.8 patients/month, with a consent rate of 69%. Protocol adherence was achieved with patients receiving > 80% of the interventions. Overall mean (SD) daily Sedation Intensity Score was 52.4 (30.3) with a mean (SD) sedation frequency of 9.75 (7.21) PRN doses per day. There was a small but statistically significant decrease in heart rate at the beginning of the music intervention. There were no study related adverse events. Eighty-eight percent of the parents thought the headphones were comfortable; 73% described their child more settled during the intervention.

**Conclusions:**

This pilot RCT has demonstrated the feasibility of a music medicine intervention in critically ill children. The study has also provided the necessary information to plan a larger trial.

**Supplementary Information:**

The online version contains supplementary material available at 10.1186/s40560-020-00523-7.

## Background

Stress induced by pain and anxiety is common in pediatric intensive care unit (PICU) patients and can impede delivery of care as well as recovery [1]. In PICU, sedation and analgesia are important not only for comfort, but also for safety [2]. Sedation and analgesia in PICU are usually achieved by using pharmacologic interventions including various narcotic and sedative medications. However, excessive use of these drugs can put patients at risk for hemodynamic/respiratory instability, prolonged ventilation, withdrawal, delirium, prolonged PICU stay and increase health care costs [2–4].

Non-pharmacologic interventions (music, noise reduction, sleep promotion, relaxation, etc.) may reduce the total requirement and associated side effects of sedation and analgesia drugs, and have been recommended by international sedation guidelines [4–6]. However, none of the guidelines state how these interventions should be provided. Non-pharmacologic measures in PICU, including music, have been inadequately studied, and the need for research on this topic has recently been identified [7–10]. In our Canadian survey, 85% of intensivists responded that non-pharmacologic interventions in PICU should be formally studied [7]. A systematic review conducted by our group found limited evidence to support or refute the use of music to reduce sedation and analgesia requirements in critically ill adults, and no evidence in PICU patients [9]. The aim of the MUSiCC pilot trial was to determine the feasibility of a pediatric music medicine trial. We hypothesized that an RCT of music medicine in critically ill children would be feasible. Further, we aimed to collect pediatric data on sedation and analgesia requirements, which will be necessary to calculate the sample size for a future, larger, trial.

## Materials and methods

The MUSiCC trial was a three-arm parallel RCT examining the use of music for sedation in PICU. A three-group design with music, noise cancellation and control groups was based on adult data showing that noise cancellation alone can reduce sedation requirements as well as pediatric evidence that noise levels are associated with sedation requirements [11, 12]. The study included children admitted to the Stollery Children’s Hospital PICU/PCICU, aged 1 month to 16 years and receiving invasive mechanical ventilation for > 24 h, and within 48 h of admission [13]. The exclusion criteria used and details on units characteristics can be found in Supplementary Material I. There were no significant changes to the study design after commencement [13].

At baseline, the following variables were recorded: demographics, ICU of admission, operative status, Pediatric Risk of Mortality score III (PRISM-III) and sedation and/or analgesia drugs use prior to ICU admission. At the time of enrollment, we collected the following variables: Pediatric Logistic Organ Dysfunction score (PELOD-2), inotrope score and need for invasive procedures (i.e. presence of invasive lines and tubes). Variables were recorded in an anonymized database using REDCap, Research Electronic Data Capture [14].

### Randomization procedure and treatment allocation

**Fig. 1:**
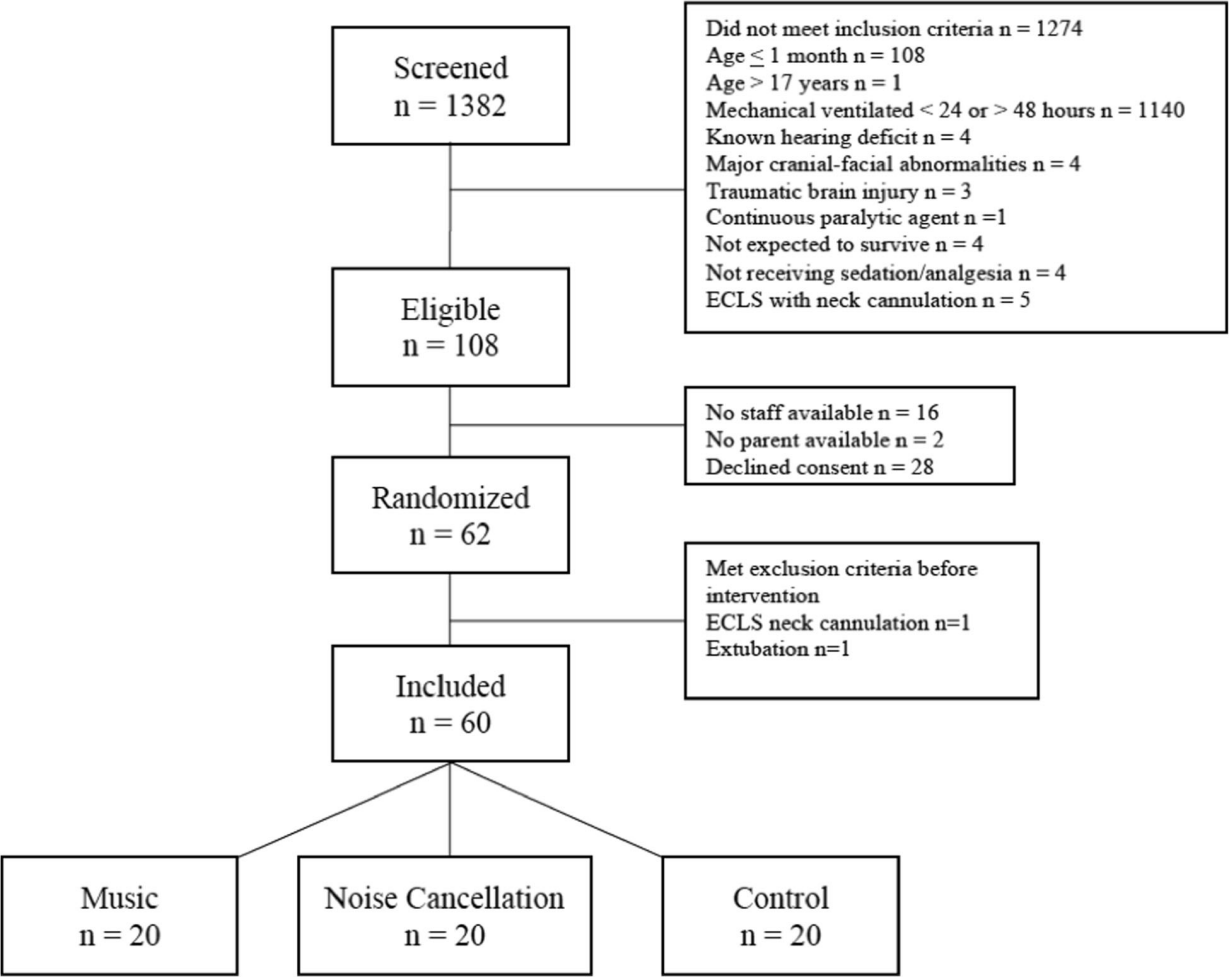
Flow chart

Subjects were identified by screening and approached for consent after their admission to the ICU. Randomization was done by a computer-based program to ensure allocation concealment and was performed by the Epidemiology Coordinating and Research Centre at the University of Alberta. A total of 60 patients were consecutively randomly assigned in a 1:1:1 ratio to music, noise cancellation or control groups (Fig. 1). In order to blind the intervention, the research nurse provided a portable music player (Apple iPod^TM^ touch, CA, USA) with music or silent recording based on group allocation and did not disclose this information to the healthcare team or the family. The iPods assigned to the noise cancellation group had a sham playlist with a silent recording that displayed on the iPod screen as if music was being played. Each 30-min playlist (music and sham) started with 1 min of silence to help maintain blinding. The iPod volume was set at 45–55 dB(A), and nurses were instructed to not modify this parameter. Based on the nature of the intervention, it was impossible to blind the use of headphones vs. control. However, outcome data was determined from the electronic medical records, blinded to group allocation.

After randomization, patients were started on the assigned intervention (music/noise cancellation/control) within 24–48 h after admission to ICU. In the music and noise cancellation groups, the intervention was delivered three times a day for 30 min at a time. The bedside nurse determined the exact time of each intervention so as not to interfere with care, within the following time windows: 7 A.M.–12 P.M. (morning intervention), 12 P.M.–4 P.M. (afternoon intervention) and 4 P.M.–8 P.M. (evening intervention). The control group received usual care. Music was delivered with the use of noise cancellation headphones (PURO® Sound Labs Kids BT2200 and BT5200, CA, USA) and an iPod touch. Pre-recorded music was selected by a music therapist and consisted of short pieces of classical music with tempos of around 60 beats per minute, preference for major keys and avoidance of dramatic moments, unsettling chords and dissonant minor keys, which can be associated with sadness [15, 16]. The decision on timing, duration and frequency of the intervention was based on limited available evidence [9, 11, 17, 18]. We created four different music playlists of 30 min each to add variation to the intervention. In the noise cancellation group, the intervention was provided with the same headphones connected to an iPod with a sham playlist with silent recording as described above. Children were assessed with the State Behavior Scale (SBS) during the intervention [19]. Signs of agitation and/or an increase in the SBS by two points indicated intolerance to the intervention. Patients were to remain on protocol as long as they received invasive mechanical ventilation or for a maximum of 7 days, whichever came first.

Other than the interventions, clinical care, including sedation and analgesia management, was not protocolized and ordered by physicians according to usual management. Assessment of the patients’ sedation status, pain and withdrawal symptoms was conducted at least every 6 h as part of nursing routine care using the SBS, Face Legs Activity Cry Consolability (FLACC) and Withdrawal Assessment Tool-1 (WAT-1) scores [19–21].

### Outcome measures

The primary outcomes of this trial were feasibility and to obtain information on sedation and analgesia requirements and variability. In order to determine feasibility, we collected information on number of eligible patients, number of patients enrolled, consent rate, time to enroll 60 patients, protocol adherence and reasons for protocol deviation. Feasibility was defined as a protocol adherence rate of ≥ 80% and a consent rate of ≥ 70% with an average patient enrollment of 5 per month. Protocol adherence was defined as receiving the allocated intervention for 30 min, 3 times/day while the patient remained in the study.

Information on sedation and analgesia requirements will allow the appropriate sample size calculations for a larger trial. Our group found that reduction in sedation requirements is a meaningful and clinically relevant outcome for a trial on non-pharmacological interventions in PICU [7]. Sedation and analgesia drug requirements were captured as a daily Sedation Intensity Score and as-needed intermittent dose (PRN) frequency [11, 22]. The Sedation Intensity Score aggregates the amount of sedation and analgesia from different drug classes using a weight-adjusted dose of each sedative administered during 4-h time blocks [11, 22]. Every sedation amount for each drug is then placed in quartiles created by using the data from all patients enrolled in the study. The values are then summed over the six 4-h blocks to obtain the daily score; higher scores indicate greater sedative exposure. Sedation frequency was captured by the daily number of PRN doses of any of the sedative and analgesia drugs given [11, 22].

This study also explored the effects of music on ICU delirium. Delirium was assessed twice a day with the Cornell Assessment of Pediatric Delirium (CAPD) instrument [23]. Patients with a score > 9 on two consecutive measurements were considered to have PICU delirium. Vital signs including heart rate, systolic blood pressure, diastolic blood pressure, respiratory rate and oxygen saturation were collected before (baseline), at 15 min, immediately after and 30 min after each intervention. Adverse events such as intolerance to the intervention and skin and/or ear problems (e.g. pressure injuries) thought to be associated with the use of headphones were monitored. Duration of invasive mechanical ventilation, PICU stay, hospital length of stay and PICU mortality were also recorded.

As part of our family centered care approach, we included parents’ perspective on the use of music for sedation in critically ill children. Parents’ opinions on the blinded intervention were explored with a survey (Supplementary Material II).

The study was approved by the University of Alberta Health Research Ethics Board (Pro00073775). Written informed consent was given by the parents/legal guardians. The study was registered at ClinicalTrials.gov (NCT03497559, https://clinicaltrials.gov/ct2/show/NCT03497559?cond=MUSIC&draw=4&rank=24).

### Statistical analysis

Assuming a protocol adherence of 80%, we calculated that a sample size of 60 patients was needed to estimate the proportion within 10% of the true rate with 95% confidence. Also, 20 participants per group followed the recommended rules for pilot trial sample size when the standardized effect size is unknown but expected to be small [24]. Baseline characteristics are presented by descriptive statistics; comparison of these characteristics among groups was done using Kruskal-Wallis test for continuous variables and Fisher exact test for categorical variables. Analysis was conducted by intention to treat. To analyze the effects of the interventions on sedation and vital signs, linear mixed-effects models were used with random intercept, to accommodate the correlation and inconstant variance between sedation requirements measurements among various time points. The model contains only time as a covariate using unstructured covariance, where variance and covariance values are estimated uniquely from the data. Linear and logistic regression models were used to analyze the effect of the interventions on mechanical ventilation, length of stay and survival. Data was analyzed with R software version 3.6.1 (Foundation for Statistical Computing, Vienna, Austria). We considered statistical significance at a *p* value ≤ 0.05.

## Results

Sixty patients (20 per group) were enrolled between March 2018 and April 2019. Demographic and baseline characteristics of study participants are displayed in Table 1. The mean (SD) age of participants was 2.0 (3.4) years, with a mean (SD) weight of 10.6 (11.1) kilograms. Thirty-five (58%) of the participants had a cardiac diagnosis and 36 (60%) were admitted after a surgical intervention. Sixteen (26%) children received sedation/analgesia drugs prior to PICU admission. Despite randomization, children assigned to the music group were younger and had higher PRISM-III scores.

**Table 1:** Baseline characteristics

Characteristic	Control *n* = 20	Music *n* = 20	Noise cancellation *n* = 20
Age—years^a^	2.02 (3.5)	1.16 (3.5)	2.02 (3.5)
Weight—kilograms^a^	12.05 (14.54)	7.22 (14.54)	12.05 (14.54)
Sex—male^b^	9 (45%)	13 (65%)	9 (45%)
PRISM Score^a^	6.65 (4.94)	8.45 (4.94)	6.65 (4.94)
Inotrope Score on admission^a^	4.08 (3.83)	10.2 (3.83)	4.08 (3.83)
PELOD Score on enrollment^a^	6.45 (1.79)	7 (1.79)	6.45 (1.79)
Type of ICU			
PCICU^b^	10 (50%)	15 (75%)	12 (60%)
PICU^b^	10 (50%)	5 (25%)	8 (40%)
Sedation prior to ICU—yes^b^	7 (35%)	4 (20%)	5 (25%)
Post-operative—yes^b^	13 (65%)	12 (60%)	11 (55%)
Cardiac diagnosis—yes^b^	10 (50%)	13 (65%)	12 (60%)
Diagnosis^b^			
Cardiac arrest	1 (5%)	0 (0%)	0 (0%)
Gastrointestinal	2 (10%)	0 (0%)	1 (5%)
Post-operative	10 (50%)	12 (60%)	10 (50%)
Respiratory	3 (15%)	7 (35%)	4 (20%)
Shock	2 (10%)	1 (5%)	1 (5%)
Trauma	1 (5%)	0 (0%)	1 (5%)
Other	1 (5%)	0 (0%)	3 (15%)
Arterial line—yes^b^	17 (85%)	18 (90%)	17 (85%)
Central line—yes^b^	18 (90%)	18 (90%)	18 (90%)
Chest tube—yes^b^	11 (55%)	11 (55%)	10 (50%)
Mediastinal tube—yes^b^	9 (45%)	9 (45%)	6 (30%)

*PRISM* pediatric risk of mortality, *PELOD* pediatric logistic organ dysfunction, *ICU* intensive care unit, *PCICU* pediatric cardiac intensive care unit, *PICU* pediatric intensive care unit

^a^Mean (SD)

^b^*n* (%)

### Feasibility

The average enrollment rate was 4.8 patients/month, with 69% of the approached parents/guardians giving consent to participate. Protocol adherence was achieved, with patients receiving a total of 358 study interventions, representing 83% (95% CI: 79–86%) of the protocolized interventions. The main reasons for missing an intervention (*n* = 74) were use of paralytic agents 28/74 (38%), parental request 9/74 (12%) and unknown cause 12/74 (16%). Only 19 (5%) interventions lasted < 30 min, with the reasons for a shorter intervention being an increase of > 2 points in the SBS 7/19 (37%), hemodynamic instability 5/19 (26%), need for an intervention unrelated to the study 4/19 (21%), receiving a paralytic agent 1/19 (5%), nurse thought time was completed 1/19 (5%) and unknown 1/19 (5%). There were no study related adverse events.

### Sedation and analgesia requirements

The overall mean (SD) daily Sedation Intensity Score for the study population was 52.4 (30.3) with a mean (SD) sedation frequency of 9.75 (7.21) PRN doses per day. There was no significant difference in mean Sedation Intensity Score and sedation frequency between groups (Table 2). The control group had a mean (SD) Sedation Intensity Score 47.6 (26.0) vs. music group 53.7 (36.9) and noise cancellation group 55.6 (26.1), *p* value = 0.561. The sedation frequency mean (SD) was also similar across groups with the control group receiving 8.58 (6.11) vs. music group 9.75 (7.1) and noise cancellation group 10.9 (8.14), *p* value = 0.511. Mean (SD) sedation, analgesia, and delirium scores were also not different across groups (Table 2). Mean (SD) WAT-1 scores were slightly higher in the music group 1.85 (1.54) vs. control 1.12 (1.17) group, *p* value = 0.020, with no significant difference between the control and noise cancellation group (Table 2). Sedation and analgesia requirements are presented in Table 3.

**Table 2:** Mixed-effects model analysis for sedation, pain, withdrawal and delirium

Variables	Control Mean (SD)	Music Mean (SD)	Mixed-effects model Effect size (95% CI)	*P* value	Noise cancellation Mean (SD)	Mixed-effects model Effect size (95% CI)	*P* value
Sedation intensity Score/day	47.6 (26.0)	53.7 (36.9)	7.08 (− 7.56, 21.73)	0.340	55.6 (26.1)	6.63 (− 8.02, 21.25)	0.371
Sedation frequency/day	8.58 (6.11)	9.75 (7.10)	1.43 (− 1.71, 4.58)	0.368	10.9 (8.14)	1.71 (− 1.45, 4.84)	0.282
SBS	− 0.76 (0.87)	− 0.74 (0.95)	0.12 (− 0.32, 0.56)	0.595	− 0.53 (0.96)	0.06 (− 0.37, 0.50)	0.772
FLACC	1.30 (1.36)	1.17 (1.26)	− 0.08 (− 0.71, 0.55)	0.796	1.62 (1.72)	0.10 (− 0.53, 0.73)	0.751
WAT-1	1.12 (1.17)	1.85 (1.54)	0.82 (0.15, 1.49)	0.020	1.65 (1.14)	0.48 (− 0.19, 1.14)	0.166
CAPD	12.47 (4.56)	13.09 (5.54)	1.18 (− 1.45, 3.81)	0.384	13.86 (4.66)	1.70 (− 0.86, 4.27)	0.199

*SBS* State Behavioral Scale (scale range from − 3 to + 2 with higher score indicating more agitation), *FLACC* Face Legs Activity Cry Consolability scale (scale range from 0 to 10 with higher score indicating higher pain), *WAT-1* Withdrawal Assessment Tool (scores range from 0 to 12 with higher score indicating more withdrawal symptoms), *CAPD* Cornell Assessment of Pediatric Delirium (score ranges from 0 to 32 with higher score indicating higher risk of delirium)

**Table 3:** Sedation and analgesia drug use composing the Sedation Intensity Score by group

Drugs	Overall *n*(%)	Music *n*(%)	Noise cancellation *n*(%)	Control *n*(%)
Chloral hydrate	118(10%)	47(12%)	45(11%)	26(8%)
Clonidine	16(1%)	8(2%)	4(1%)	4(1%)
Dexmedetomidine	164(14%)	45(11%)	69(18%)	50(15%)
Fentanyl	114(10%)	47(12%)	32(8%)	35(10%)
Hydromorphone	234(21%)	78(19%)	80(20%)	76(22%)
Ketamine	125(11%)	47(12%)	37(9%)	41(12%)
Lorazepam	91(8%)	32(8%)	32(8%)	27(8%)
Midazolam	166(15%)	55(14%)	61(15%)	50(15%)
Morphine	30(3%)	17(4%)	6(2%)	7(2%)
Propofol	81(7%)	27(7%)	28(7%)	26(8%)

### Vital signs before, during and after the music and noise cancellation interventions

There was a statistically significant decrease in heart rate at the beginning of the music and noise cancellation interventions compared to baseline (Table 4). After noise cancellation, the respiratory rate also decreased compared to baseline. There were no significant differences in blood pressures or oxygen saturations before, during and after the interventions (Table 4).

**Table 4:** Mean (SD) vital signs before, during and after the intervention by group

Variables	Prior to the intervention	15 min of the intervention	% of change from prior to the intervention	The end of the intervention	% of change from prior to the intervention	30 min after the intervention	% of change from prior to the intervention	*P* value
Music								
HR/minute	122 (23.3)	119 (25.2)*	1.84	120 (24.7)	1.46	121 (25.6)	0.54	0.004
RR/minute	27.2 (7.7)	27.0 (7.65)	0.73	27.2 (9.29)	0.22	27.9 (8.74)	2.50	0.631
SBP—mmHg	88.3 (13.1)	88.6 (12.91)	0.33	87.4 (13.4)	0.96	87.8 (13.8)	0.59	0.438
DBP—mmHg	49.9 (9.62)	49.6 (9.41)	0.58	48.9 (9.75)	2.02	49.4 (9.69)	1.10	0.306
O_2_ saturation—%	92.9 (7.92)	92.7 (8.24)	0.18	91.7 (11.1)	1.23	92.8 (8.39)	0.11	0.378
Noise cancellation								
HR/minute	126 (23.3)	124 (21.6) *	1.54	124 (21.5)	1.20	125 (22.1)	1.15	0.021
RR/minute	27.2 (6.58)	25.5 (6.03) *	6.30	26.6 (6.76)	2.27	27 (8.22)	1.03	0.001
SBP—mmHg	85.6 (13.7)	82.9 (11.6)	3.21	83.9 (13.1)	2.04	84.6 (11.6)	1.26	0.112
DBP—mmHg	48.3 (9.72)	47.2 (8.12)	2.17	47.5 (9.04)	1.57	47.8 (9.64)	0.97	0.265
O_2_ saturation—%	93.6 (7.84)	93.7 (7.39)	0.13	93.8 (7.16)	0.15	93.7 (7.47)	0.13	0.481

*HR* heart rate, *RR* respiratory rate, *SBP* systolic blood pressure, *DBP* diastolic blood pressure

*P value = 0.008 compare to the “Prior to the intervention value”

### Other outcomes

Mechanical ventilation days, length of stay and survival are shown in Table 5.

**Table 5:** Outcome variables by group

Variables	Control *n* = 20	Music *n* = 20	Noise cancellation *n* = 20	*P* value
Mechanical ventilation—days^a^	7.3 (5.49)	8.2 (5.49)	7.3 (5.49)	0.723
ICU LOS—days^a^	11.1 (8.33)	16.0 (8.33)	11.1 (8.33)	0.145
Hospital LOS—days^a^	39.6 (47.0)	59.2 (47.0)	39.6 (47.0)	0.585
Survival to hospital discharge, *n* (%)—yes	18 (90%)	17 (94.5%)	19.0 (95%)	0.999

*ICU* intensive care unit, *LOS* length of stay

^a^Mean (SD)

### Parent survey

Eighteen (70%) of the respondent parents thought the intervention was useful during their child’s ICU admission. Sixteen (62%) thought the intervention reduced their child’s anxiety, while 9 (35%) thought it helped to reduce pain. However, only 11 (42%) perceived that the intervention helped to reduce the need for sedatives and analgesics. The majority of the parents, 23 (88%), thought the headphones were comfortable. The majority, 19 (73%) described their child’s reaction during the intervention as “more settled and asleep”; however, 3 (11%) of the parents thought their child became more agitated during the intervention.

## Discussion

While music appears to be a promising intervention, there is presently no evidence that it decreases use of pharmacologic therapies for sedation and analgesia in critically ill children [9]. A pilot RCT is a necessary step toward the conduct of a definitive music medicine intervention trial in critically ill children. This study is also needed to allow formal sample size calculations for a future larger trial. Our MUSiCC trial demonstrated the feasibility of a music and a noise cancellation intervention in the PICU/PCICU environment. Despite having consent (69%) and enrollment rates (4.8 patients/month) slightly below the pre-specified feasibility thresholds, the study was well accepted and patients received > 80% of the protocolized interventions. A higher enrollment rate could have been achieved by including patients on non-invasive mechanical ventilation. Missed interventions were mainly due to the use of paralytic agents around the times of interventions. A more flexible schedule of interventions or the option of using the intervention as PRNs may help to address this issue in a larger trial. Only 9 (2%) of the interventions were not conducted based on parental request. These requests were based on the concern of their children being too sick rather than the belief that the intervention was harmful or causing distress. The most common impression from parents was that their children were more settled and asleep during the interventions supporting the use of music. Our results showed that sedation, analgesia, and delirium scores were not statistically different across groups. There was also a statistically significant (but likely clinically irrelevant) difference in the WAT-1 scores between the music and the control groups. The wide 95% CIs in the mean differences in sedation requirements between groups presented in Table 2 demonstrates the results were compatible with an effect size in that interval and, hence, include the possibility of benefit or harm from the intervention. We observed a decrease in HR after the music was started. However, absolute and proportion of change in vital signs were small. A larger multicenter RCT with age stratification will avoid these problems. Based on our results, a future trial aimed to demonstrate a 20% reduction in daily Sedation Intensity Score with an alpha of 0.05 and power of 0.8 will need to enroll 119 patients/group; a more conservative approach with an alpha of 0.005 and same power will require 201 children/group.

This is the first study to use Chlan’s Sedation Intensity Score in the PICU environment [11, 22]. The Sedation Intensity Score allows aggregation of all the different sedation and analgesia drugs given despite the inability to calculate equivalent doses for drugs of different classes. In recent years, it has been recognized that over-sedation not only puts patients at risk for hemodynamic/respiratory instability, but also for prolonged ventilation, withdrawal, delirium and the inability to mobilize critically ill patients leading to longer times for recovery [2, 25]. In this context, a goal directed strategy establishing daily goals of sedation and analgesia has been implemented across ICUs and is known as the “ICU liberation” strategy [25]. Hence, sedation and analgesia scores are utilized not only to assess the patients’ level of pain and sedation, but also to establish goals as part of the daily care plan. Because of this, pain and sedation scores cannot be the primary outcome of trials looking at the effect of non-pharmacologic interventions as drugs are titrated to target a specific score appropriate to the patient’s condition. A reduction in sedation/analgesia drugs requirements has been identified in as a meaningful clinical outcome for trials investigating new sedation and analgesia strategies [7].

Although music has been used for years in healthcare, the exact mechanisms by which it can reduce pain/anxiety are not well understood. It is known that music can modify emotional status by releasing anti-stress hormones and by activating the limbic system of the brain [26]. According to the gate control theory of pain, distractions such as music can block certain neural pathways and diminish the amount of perceived pain [26, 27]. Studies using music in mechanically ventilated adults found that music was associated with lower levels of anxiety, lower sedation requirements [11, 27]. In pediatrics, music has been shown to reduce procedural pain and anxiety in a variety of clinical settings, but these studies used music for distraction and did not include critically ill children [8, 28–30]. In newborns, music has been shown to be effective in reducing pain and stress behaviours during procedures and has also been associated with more stable vital signs, better weight gain, shorter length of stay and increased parental satisfaction [31–33]. The evidence for the use of music in the PICU is very limited and does not include studies assessing the impact of music on sedation and analgesia requirements [9]. To our knowledge, there has been only one RCT evaluating the effects of music on vital signs and pain scores in critically ill children [34]. While results were positive, this trial did not assess sedation requirements. Two recently published pilot trials used music interventions in PICU [10, 35]. Rennick et al. used music at the end of a soothing (touch and reading) intervention [34]. One hour of music was thought by parents to calm their children; however, details on the type music and effects on sedation requirements were not reported. On the other hand, Liu et al. investigated the effect of music on sedation scores, vital signs and midazolam utilization [10]. Data on analgesia and other sedatives was not reported. However, these two pilot studies add to the evidence that a music intervention in PICU is well accepted by parents and the health care team.

Our trial differs from previous studies looking at the use of music in ICU in several important aspects. Studies in critically ill children have most often been limited to premature newborns who were neither on mechanical ventilation nor on sedatives [31–33]. Patients included in critically ill adult trials were relatively stable and the majority were in a weaning phase from their mechanical ventilation [11, 36–40]. None of the studies evaluated heavily sedated patients in the acute phase of their illness; the included patients were on sedatives for some time, with variability in length of stay at the time of study entry. Ideally, if non-pharmacologic interventions can reduce the use sedation and their side effects, they should be implemented early in the patient’s admission. This approach has significant challenges since in PICU most patients will not be able to select their own music or decide when they would like the intervention to take place. Including parents in the music selection may help to overcome this barrier [10]. However, the involvement of parents in the music selection should be guided by a music therapist to assure the selection is in line with the objectives of the intervention.

This pilot RCT has the following strengths. First, this RCT explored the use of music for sedation in mechanically ventilated critically ill children in the acute phase of their illness. Second, this pilot trial was built upon a previous survey, cohort study and systematic review that provided the information necessary to determine the appropriate design and outcomes [7, 9, 12, 24]. Third, this is the first study to use a novel sedation outcome measure, the Sedation Intensity Score, in the PICU environment [11, 22]. This approach allowed us to assess the use of sedation and analgesia requirement thoroughly, which had not been achieved in previous studies. Finally, blinding the interventions allocation between the music and noise cancellation has helped to reduce bias.

This pilot RCT also has limitations. First, frequency, timing and length of the music intervention was chosen based on limited available evidence on the use of music in critically ill patients [9, 11, 17, 18]. There is limited evidence that classical music with a tempo of around 60 beats per minute and a preference for major keys can provide sedation and is appropriate for all ages [15, 16, 18, 26]. Whether other types of music or different dosing of the music intervention could be more effective for critically ill children is unknown. Second, music therapy is defined as the clinical and evidence-based use of music by a music therapist to obtain individualized goals for a certain patient or group of patients [15, 26]. Ideally, each intervention should be conducted by a music therapist who can adjust the intervention based on the patient’s response. However, the conduct of a clinical trial using live music therapy (as opposed to pre-recorded music) to reduce sedation requirements in mechanically ventilated and critically ill children would be challenging. Last, we did not use a specific sedation protocol but rather a pragmatic approach which could have influenced our results.

## Conclusion

This pilot RCT has explored the feasibility of a music medicine intervention trial in critically ill children. The study has also provided information to plan a larger trial to determine the efficacy of music to reduce sedation and analgesia requirements in PICU.

## Supplementary Information

**Additional file 1: Supplementary Material I.** Exclusion criteria.

**Additional file 2: Supplementary Material II.** Parents survey.

## Data Availability

The datasets generated and/or analyzed during the current study are not publicly available due to the pilot nature of the study but are available from the corresponding author on reasonable request.

## Abbreviations

CAPD: Cornell Assessment of Pediatric Delirium
CI: Confidence interval
FLACC: Face Legs Activity Cry Consolability
ICU: Intensive care unit
PELOD: Pediatric Logistic Organ Dysfunction
PRISM: Pediatric Risk of Mortality
PCICU: Pediatric cardiac intensive care unit
PICU: Pediatric intensive care unit
PRN: As-needed intermittent doses
RCT: Randomize controlled trial
SBS: State Behavior Scale
SD: Standard deviation
WAT: Withdrawal Assessment Tool
