# Supplementary material for: Music Use for Sedation in Critically ill Children (MUSiCC trial): a pilot randomized controlled trial
Source: J Intensive Care. 2021 Jan 12;9:7. doi: 10.1186/s40560-020-00523-7 (PMC7802123; doi:10.1186/s40560-020-00523-7)
Supplement: Supplementary file 1 — Additional file 1: Supplementary Material I. Exclusion criteria. [file 40560_2020_523_MOESM1_ESM.docx]

**Supplementary Material I: Exclusion criteria**

**-** Known hearing deficit

- Infants < 1 month old or <3 kilograms

- Major cranial-facial abnormalities

- Traumatic brain injury

- Not receiving sedation and/or analgesia drugs

- Continuous infusion of paralytic agents

- Extracorporeal Membrane Oxygenation with neck cannulation

- Expected to die in the next 48 hours

- Enrolled in another sedation intervention study

**Unit Characteristics**

- Both ICUs (PCICU and PICU) are run by pediatric intensivists.

- Both ICUs have an open policy with respect of family visits and parents are allowed in the unit 24 hours, 7 days a week.

- Sedation and analgesia drugs are indicated by physicians without using any specific sedation/analgesia protocol.

- PCICU has 16 single rooms.

- PICU, at the time of the study, had 4 single rooms and 10 beds in an open area.
