# Supplementary material for: Music Use for Sedation in Critically ill Children (MUSiCC trial): a pilot randomized controlled trial
Source: J Intensive Care. 2021 Jan 12;9:7. doi: 10.1186/s40560-020-00523-7 (PMC7802123; doi:10.1186/s40560-020-00523-7)
Supplement: Supplementary file 2 — Additional file 2: Supplementary Material II. Parents survey. [file 40560_2020_523_MOESM2_ESM.docx]

**Supplementary Material II: Parent’s Survey**

1. In your opinion, how helpful was the use of the intervention while your child was in the intensive care unit:
   1. Very helpful
   2. Somewhat helpful
   3. Neutral
   4. Not helpful
   5. Not helpful at all
   6. I don’t know
2. To what extent do you agree with the following statement: “The use of the intervention reduced my child’s **anxiety** while he/she was in the intensive care unit.”
   1. Strongly agree
   2. Agree
   3. Neutral
   4. Disagree
   5. Strongly disagree
   6. Don’t know
3. To what extent do you agree with the following statement: “The use of the intervention reduced my child’s **pain** while he/she was in the intensive care unit.”
   1. Strongly agree
   2. Agree
   3. Neutral
   4. Disagree
   5. Strongly disagree
   6. Don’t know
   7. Strongly disagree
   8. Don’t know
4. To what extent do you agree with the following statement: “The use of the intervention reduced my child’s need for **sedatives** while he/she was in the intensive care unit.”
   1. Strongly agree
   2. Agree
   3. Neutral
   4. Disagree
   5. Strongly disagree
   6. Don’t know
5. To what extent do you agree with the following statement: “The use of the intervention reduced my child’s need for **pain** medications while he/she was in the intensive care unit.”
   1. Strongly agree
   2. Agree
   3. Neutral
   4. Disagree
6. How comfortable do you think the headphones used were for your child:
   1. Very uncomfortable
   2. Uncomfortable
   3. Neutral
   4. Comfortable
   5. Very comfortable
   6. Don’t know
7. How would you describe your child’s most common reaction when the intervention was used?
   1. Was more settled and remained awake

b. Was more settled and slept

c. Was more agitated

d. Was more agitated with more crying

e. No difference

f. Don’t know

1. Would you like to leave some comments about the use of the intervention in intensive care?
